# Tuberculosis Risk Stratification of Psoriatic Patients Before Anti-TNF-α Treatment

**DOI:** 10.3389/fimmu.2021.672894

**Published:** 2021-06-03

**Authors:** Farida Benhadou, Violette Dirix, Fanny Domont, Fabienne Willaert, Anne Van Praet, Camille Locht, Françoise Mascart, Véronique Corbière

**Affiliations:** ^1^ Dermatology Department, Hôpital Erasme, Université Libre de Bruxelles (U.L.B.), Brussels, Belgium; ^2^ Laboratory of Vaccinology and Mucosal Immunity, Université Libre de Bruxelles (U.L.B.), Brussels, Belgium; ^3^ Univ. Lille, CNRS, Inserm, CHU Lille, Institut Pasteur de Lille, U1019 – UMR 8204 – CIIL - Center for Infection and Immunity of Lille, Lille, France

**Keywords:** psoriasis, Tumor necrosis factor-α inhibitors, latent tuberculosis infection, tuberculin skin tests, interferon-γ-release assays, QuantiFERON, heparin-binding haemagglutinin

## Abstract

Psoriasis is a skin inflammatory condition for which significant progress has been made in its management by the use of targeted biological drugs. Detection of latent *M. tuberculosis* infection (LTBI) is mandatory before starting biotherapy that is associated with reactivation risk. Together with evaluation of TB risk factors and chest radiographs, tuberculin skin tests (TST) and/or blood interferon-γ-release assays (IGRA), like the QuantiFERON (QFT), are usually performed to diagnose *M. tuberculosis* infection. Using this approach, 14/49 psoriatic patients prospectively included in this study were identified as LTBI (14 TST^+^, induration size ≥ 10mm, 8 QFT^+^), and 7/14 received prophylactic anti-TB treatment, the other 7 reporting past-treatment. As the specificity and sensitivity of these tests were challenged, we evaluated the added value of an IGRA in response to a mycobacterial antigen associated with latency, the heparin-binding haemagglutinin (HBHA). All but one TST^+^ patient had a positive HBHA-IGRA, indicating higher sensitivity than the QFT. The HBHA-IGRA was also positive for 12/35 TST^-^QFT^-^ patients. Measurement for 15 psoriatic patients (12 with HBHA-IGRA^+^) of 8 chemokines in addition to IFN-γ revealed a broad array of HBHA-induced chemokines for TST^+^QFT^-^ and TST^-^QFT^-^ patients, compared to a more restricted pattern for TST^+^QFT^+^ patients. This allowed us to define subgroups within psoriatic patients characterized by different immune responses to *M. tuberculosis* antigens that may be associated to different risk levels of reactivation of the infection. This approach may help in prioritizing patients who should receive prophylactic anti-TB treatment before starting biotherapies in order to reduce their number.

## Introduction

Psoriasis is a frequent skin inflammatory condition with a worldwide prevalence of 3%, characterized by erythematous and scaly plaques that may affect any part of the body (1, 2). Psoriatic patients may develop comorbidities, such as psoriatic arthritis and cardiovascular diseases, leading to the concept of a systemic immune-mediated inflammatory disease (IMID) (3). Significant progress has been made in the management of psoriasis by the use of targeted biological drugs, initially limited to tumor necrosis factor-α (TNF-α) inhibitors (4). Patients receiving TNF-α-targeted therapies have an increased risk of reactivation of a latent *Mycobacterium tuberculosis* infection (LTBI), and although there are few and discrepant specific reports in psoriatic patients (5), the risk of active tuberculosis (aTB) is, according to a recent meta-analysis, doubled for patients treated with anti-TNF-α (6). The use of biological drugs to treat psoriasis was further extended to other therapeutic agents targeting the interleukin (IL)-23/IL-17 axis (7, 8), but their potential risk of reactivation of LTBI is not yet firmly established (9).

Classically, *M. tuberculosis* infection in humans is thought to present either as aTB or as LTBI defined by the presence of immunological responses to mycobacterial antigens in absence of clinical symptoms of disease (10, 11). LTBI subjects are thought to present a life-long risk of reactivation of the infection, with 5 to 15% of them developing aTB during their lifetime (11). Recent data however challenged this concept and indicated that LTBI comprises a range of infection outcomes associated with different bacterial persistence and host containment, from cleared infection to low-grade TB (10). It became evident that these last individuals are probably more at risk to reactivate the infection compared to other LTBI subjects.

In view of the higher risk of psoriatic patients to reactivate LTBI when receiving TNF-α-targeted therapies, detection of LTBI before initiating biotherapies is mandatory and essential to provide preventive anti-TB treatment (9, 12). This detection is nowadays based on the classical definition of LTBI, e.g. on the detection of memory T cell responses to mycobacterial antigens, revealing the presence of host sensitization to these antigens (11). The tuberculin skin test (TST) is the gold standard for this detection since decades, in spite of possible false-positive results in Bacillus Calmette-Guérin (BCG)-vaccinated subjects and in non-tuberculous mycobacteria (NTM)-infected patients (13), and despite possible lower sensitivity in patients suffering from IMID with immune-suppressive treatment history (14). Therefore, TST has been replaced in several countries by interferon-gamma (IFN-γ) release assays (IGRAs). These blood tests measure the IFN-γ secretion within whole blood or by peripheral blood mononuclear cells (PBMC), upon *in vitro* stimulation with peptides from the mycobacterial antigens early-secreted antigenic target-6 (ESAT-6), culture filtrate protein-10 (CFP-10), and sometimes TB7.7 (9). These IGRAs, commercially available as the QuantiFERON (QFT) (Qiagen, Hilden, Germany) or the T-SPOT.TB (Oxford Immunotec, Oxford, United Kingdom), are more specific for *M. tuberculosis* infection than TST, as the antigens used for *in vitro* stimulation are absent from BCG and most NTM. In addition, they both include positive and negative controls to identify possible false negatives. However, they were reported by several authors to have lower sensitivity than initially thought to detect immune responses to *M. tuberculosis* antigens (14), so that in Belgium, a low TB incidence country (<10 new cases/100.000 inhabitants/year) with a low BCG vaccination coverage, IGRAs are recommended only in case of doubtful TST results, or to increase sensitivity in patients already receiving immunosuppressive drugs (www.fares.be).

Using either TST or IGRA to detect LTBI before the initiation of TNF-α-targeted agents is however not optimal as prophylactic anti-TB treatment in these selected patients did not provide them complete protection from developing aTB (15). Therefore, in addition to a careful evaluation of the patient’s risk factors for LTBI and chest X-ray radiography to exclude aTB, a dual strategy performing both tests (TST and IGRA) is now largely recommended to reduce any possible risk of developing aTB. The positivity of any of these tests for the diagnosis of LTBI should be considered (16). Unfortunately, neither the TST, nor the IGRA allowed us to detect patients with the highest risk of reactivation as they cannot differentiate the newly recognized different stages within the spectrum of LTBI and are positive both in LTBI subjects and in patients with aTB (17).

Given the limitations of the TST and the commercial IGRAs to diagnose LTBI in patients with IMID, and their inability to select among LTBI subjects those who have the highest risk to reactivate the infection, we evaluated in this study the added value of an IGRA based on the latency-associated antigen heparin-binding haemagglutinin (HBHA), reported to detect LTBI with high sensitivity and specificity (18), and we compared the results of the HBHA-IGRA to those of the TST and of the QFT.

## Material and Methods

### Study Population

Forty-nine adult patients suffering from psoriasis were prospectively recruited from the outpatient clinic of the Dermatology department at the “hôpital Erasme” as part of their evaluation before starting biotherapy (Ethics Committee 021/406, P2012/082). TB screening performed for all participants included TST (0.1 ml tuberculin PPD RT23 2 TU, SSI, Copenhagen, DK), chest X-ray, and QFT. TST were read after 72 hours and the results were assessed in the context of the patient’s individual TB risk factors. In the absence of TB risk factors, TST^-^QFT^-^ patients without chest X-Ray sign suggesting aTB, were considered as non-infected with *M. tuberculosis*. QFT^+^ and/or TST^+^ patients (induration size ≥ 15 mm) were considered as LTBI after exclusion of aTB. In the context of patients at risk to reactivate LTBI, patients with a TST positivity between 10 and 14 mm were also considered as being LTBI (www.fares.be). Four patients were treated with methotrexate at the time of inclusion. Ten others already received anti-TNF-α antibodies and were included in this study before changing their biotherapy. When they were initially evaluated for possible LTBI before their first anti-TNF-α treatment, 3/10 were considered LTBI and received at that time prophylactic anti-TB treatment.

### QuantiFERON-TB Gold

QFT (QuantiFERON-TB Gold In-tube) was performed according to the manufacturer instructions (www.qiagen.com). A positive QFT test was defined as ≥ 0.35 IU/ml IFN-γ released in response to the mycobacterial peptides, after subtraction of the concentration obtained for the unstimulated condition, with a result > 25% of the unstimulated condition.

### HBHA-IFN-γ Release Assay (IGRA)

PBMC were isolated from fresh blood samples and *in vitro* stimulated during 24 hours at 37°C under 5% CO_2_ with 2 µg/ml HBHA, left unstimulated in culture medium (negative control) or were stimulated with 0.5 µg/ml staphylococcal enterotoxin B (SEB, Sigma-Aldrich, Bornem, Belgium) (positive control). IL-7 was added in the culture medium at 1 ng/ml to increase the sensitivity of the 24 hrs assay (19). HBHA was purified from *Mycobacterium bovis* BCG culture supernatants by heparin-Sepharose chromatography (Sepharose CL-6B; Pharmacia LKB, Piscataway, NJ) (20). The bound material was eluted by a 0-500 mM NaCl gradient and was further passed through a reverse-phase high-pressure liquid chromatography (HPLC; Beckman Gold System), using a Nucleosil C18 column (TSK gel Super ODS; Interchim) equilibrated in 0.05% trifluoroacetic acid. Elution was performed by a linear 0-80% acetonitrile gradient and HBHA eluted at 60% acetonitrile (21). The HPLC chromatogram revealed a single peak and analysis by SDS-PAGE showed a single band after Coomassie-blue staining, indicating the absence of contamination of HBHA with other proteins.

Cell culture supernatants were frozen at -20°C until measurement of secreted cytokines/chemokines. IFN-γ concentrations were measured by ELISA (19). IFN-γ concentrations < 50 pg/ml in the non-stimulated condition and > 200 pg/ml in the positive controls were required for further analysis of the results. When detectable, IFN-γ concentrations obtained under non-stimulated conditions were subtracted from those obtained in response to HBHA. A positive HBHA-IGRA was defined as IFN-γ concentrations ≥ 50 pg/ml IFN-γ as previously determined by ROC curve analysis comparing results obtained for LTBI subjects to those of non-infected controls (19).

### Multiple Cytokine/Chemokine Measurements

Based on our previous experience with *M. tuberculosis-*infected patients, 8 cytokines/chemokines were measured in addition to IFN-γ in the 24 h culture supernatants of HBHA-stimulated PBMC from 12 HBHA-IGRA^+^ psoriatic patients and from 3 HBHA-IGRA^-^ patients taken as negative controls: granulocyte macrophage colony-stimulation factor (GM-CSF), IFN-γ, IL-1β, IL-2, IL-6, IL-10, IL-17A, macrophage inflammatory protein (MIP-1α), and TNF-α. The cytokine/chemokine concentrations were measured by Milliplex human cytokine/chemokine kits (Merck, Belgium) according to the manufacturer’s instructions with supernatants dilution factors specific for each analyte to obtain concentrations within the standard curves. Results were analyzed with a Bio-Plex^®^ MAGPIX™ Multiplex reader, Bio-Plex Manager™ MP Software and Bio-Plex Manager 6.1 Software (BIO-RAD laboratories, Nazareth Eke, Belgium). When detectable, the analyte concentrations in the antigen-free conditions were subtracted from those obtained with antigen stimulation. Concentrations below the detection limit were allocated an arbitrary value of 5 pg/ml, whilst results exceeding the assay’s upper limit of detection were attributed the concentration corresponding to this limit. For each marker, the positivity limit was arbitrarily determined as being minimum 4 times the detection limit or maximum 2 times the median concentration obtained for non-infected patients when cytokines/chemokines were detectable. A grey zone of doubtful positivity defined as ± 20% of the cut-off value was established for each analyte. A scale representing the intensity of cytokine/chemokine concentrations was established for each analyte from negative values to doubtful, low and strong cytokine/chemokine concentrations.

### Statistical Analysis

Differences between several groups were assessed by the non-parametric Kruskal-Wallis test, followed by the non-parametric Dunn test. Differences between HBHA-induced IFN-γ concentrations at two different time-points were evaluated by the paired Wilcoxon test. A value of *p <*0.05 (*) was considered significant. All results were obtained with the Graphpad Prism Software version 4.0.

## Results

### Prevalence of *M. tuberculosis* Infection in Psoriatic Patients According to Standard Criteria

**Figure 1:**
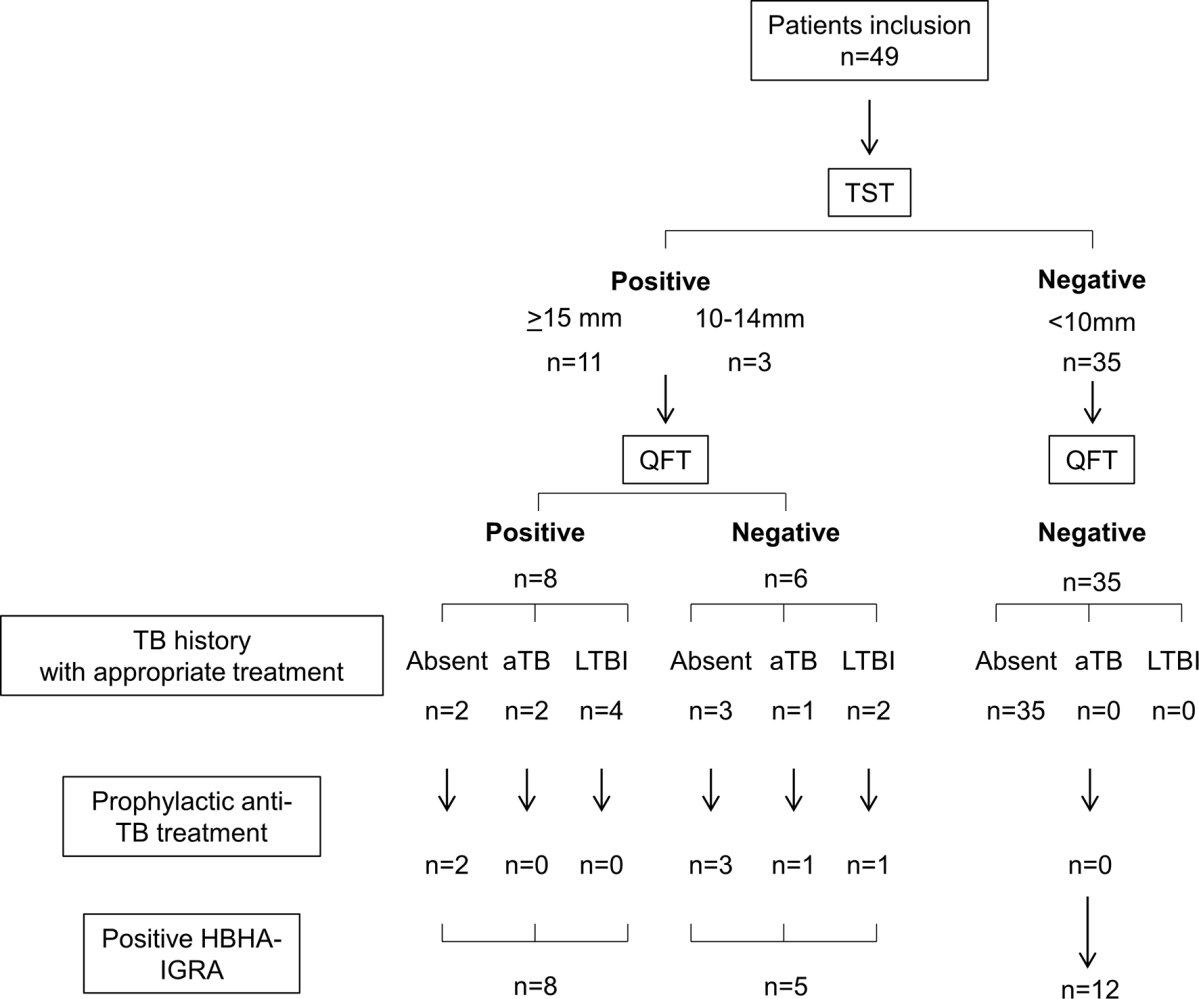
Algorithm of the patients’ classification. n, number of patients; TST, tuberculin skin test; QFT, QuantiFERON TB Gold In tube; TB, tuberculosis; HBHA-IGRA, Heparin-binding haemagglutinin-interferon-gamma release assay.

**Figure 2:**
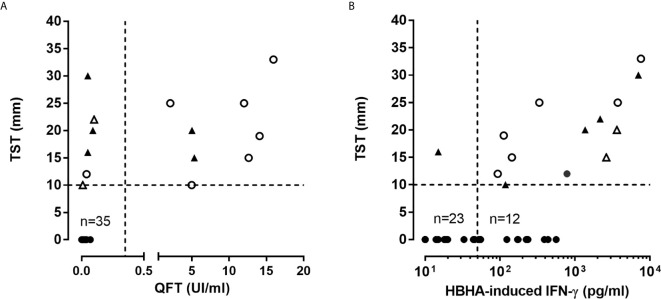
Correlation between immunoassays performed in psoriatic patients. **(A)** TST and QFT results were compared in all patients. Triangles represent patients who received anti-TB prophylaxis, open symbols represent patients with a past anti-TB treatment. **(B)** TST and HBHA-IGRA results were compared in all patients. Triangles represent patients who received anti-TB prophylaxis and open symbols (triangle or circle) represent QFT^+^ patient. TST results are given as the size of induration in mm, QFT results are expressed in international unit (UI) of IFN-γ par ml of blood, and HBHA-IGRA results are reported as concentration of IFN-γ (pg/ml) released in 24 h culture supernatants of PBMC incubated with HBHA.

In Belgium, a low-TB incidence country, forty-nine adult patients suffering from psoriasis were prospectively recruited from the outpatient clinic of the Dermatology department (hôpital Erasme), as part of their evaluation before starting biotherapy. The main demographic and clinical characteristics of these patients are reported in **Table 1**. Eleven patients had a positive TST≥15 mm and, in the absence of clinical and/or radiological signs of aTB, were classified as LTBI. Three had a TST induration size between 10 and 14 mm, and in the context of a future biotherapy, they were considered as LTBI as recommended in Belgium, and 35 patients had a negative TST (**Figure 1**). TST results were probably not influenced by previous BCG vaccination recorded for 2/14 TST^+^ and for 4/35 TST^-^ patients (**Table 1**). To avoid possible false negative TST results in patients with abnormal cellular immune responses due to their pathology and/or their treatment, QFT was performed on all patients, as now largely recommended. The QFT was positive for 8/49 patients, all of them having a positive TST (≥10 mm) (**Figure 1**). TST and QFT results were not correlated (**Figure 2A**), and the presence of LTBI risk factors was higher in the QFT^+^ (6/8 = 75%) than in the QFT^-^ (3/6 = 50%) LTBI patients (**Table 1**). Altogether, this resulted in a pre-selection of patients for prophylactic anti-TB treatment of 14/49 patients (28.6%).

**Table 1:** Demographic and clinical characteristics of the study population.

	TST^+^			TST^−^	
	QFT^+^HBHA-IGRA ^+^n=8	QFT^−^HBHA-IGRA ^+^n=5	QFT^−^HBHA-IGRA^-^n=1	QFT^−^HBHA-IGRA ^+^n=12	QFT^−^HBHA-IGRA^− ^n=23
**Age (years), median (range)**	52 (34–82)	41 (34–51)	61	44 (35–54)	50 (25-67)
**Male, *n* (%)**	8 (100)	4 (80)	0 (0)	6 (50)	14 (61)
**PASI, median (range)**	10 (7-15)	12 (10-27)	11	12 (8-27)	12 (5-33)
**Ethnic origin, *n (%)***					
Caucasian	6 (75)	4 (80)	/	10 (83)	18 (78)
African	2 (25)	1 (20)	/	2 (17)	5 (22)
Asian	/	/	1 (100)	/	/
**BCG vaccination, *n (%)***					
No	7 (87)	2 (40)	1	11 (92)	19 (83)
Yes	1 (13)	1 (20)	/	/	4 (17)
Unknown	0	2 (40)	/	1 (8)	/
**Risk factors for LTBI/aTB, *n (%)***					
High TB incidence country (Birth, Travel)	4 (50)	1 (20)	1 (100)	1 (8)	2 (9)
Contact or possible contact with TB patients (family/work)	2 (25)	/	/	1 (8)	3 (13)
Chest X-ray suggestive of previous TB	1* (12.5)	1 (20)	/	/	/
**TB History, *n (%)***					
aTB	2 (25)	1 (20)	/	/	/
LTBI	4 (50)	2 (40)	/	/	/
**Ongoing psoriasis treatment, *n* (%)**					
Adalimumab	2 (25)	/	/	/	3 (13)
Etanercept	/	1 (20)	/	3 (25)	1 (4.3)
Methotrexate	1 (12.5)	/	1 (100)	/	2 (9)

TST, Tuberculin Skin Test; QFT, QuantiFERON TB Gold In tube; HBHA, Heparin-binding haemagglutinin; IGRA, Interferon gamma release assay; n, number; PASI, Psoriasis Area Severity Index; BCG, Bacille Calmette-Guérin; LTBI, Latent Tuberculosis Infection; aTB, active Tuberculosis; *patient with 2 risk factors: he was born and travel in high TB incidence country and had chest X-ray suggestive of previous aTB.

Thirty-eight/49 included patients received anti-TNF-α (n=29) or anti-IL-23/12 (n=9) antibodies after inclusion in this study, and none of them developed aTB during a 2 year follow-up. An alternative therapeutic option was chosen for the other 11 patients. Among the 14 patients pre-selected for an initial prophylactic anti-TB treatment, 7 did not receive it because they reported a past-treatment for LTBI (n=5, less than 5 years before their inclusion), or for aTB (n=2 without radiological sequela) (**Figures 1** and **2A** with open symbols for patients with a past treatment).

### Added Value of the HBHA-IGRA

As the QFT was recently reported to be less sensitive to detect LTBI subjects than previously thought, even in a healthy population (14), and as the sensitivity of the HBHA-IGRA was reported to be higher than that of the QFT and to help to stratify LTBI subjects in different subgroups (18, 22), we evaluated the sensitivity of the HBHA-IGRA to detect *M. tuberculosis* infection in this cohort of psoriatic patients. Among the 14 TST^+^ patients, 13 of them had a positive HBHA-IGRA result, indicating that the HBHA-IGRA were better correlated with the TST than the QFT (**Figures 1** and **2B**). The only TST^+^ patient with a negative HBHA-IGRA was a patient with a TB risk factor (nurse) on immunosuppressive treatment (methotrexate), with a TST induration size of 16 mm in spite of a negative QFT (**Figure 2B**). Among the 13 TST^+^ HBHA-IGRA^+^ patients, only 8 had a positive QFT (represented by open symbols on **Figure 2B**). The results of the HBHA-IGRA were not considered for the decision to provide or to avoid prophylactic anti-TB treatment, as this test was still under investigation in these potentially immunocompromised patients (**Figure 1**).

**Figure 3:**
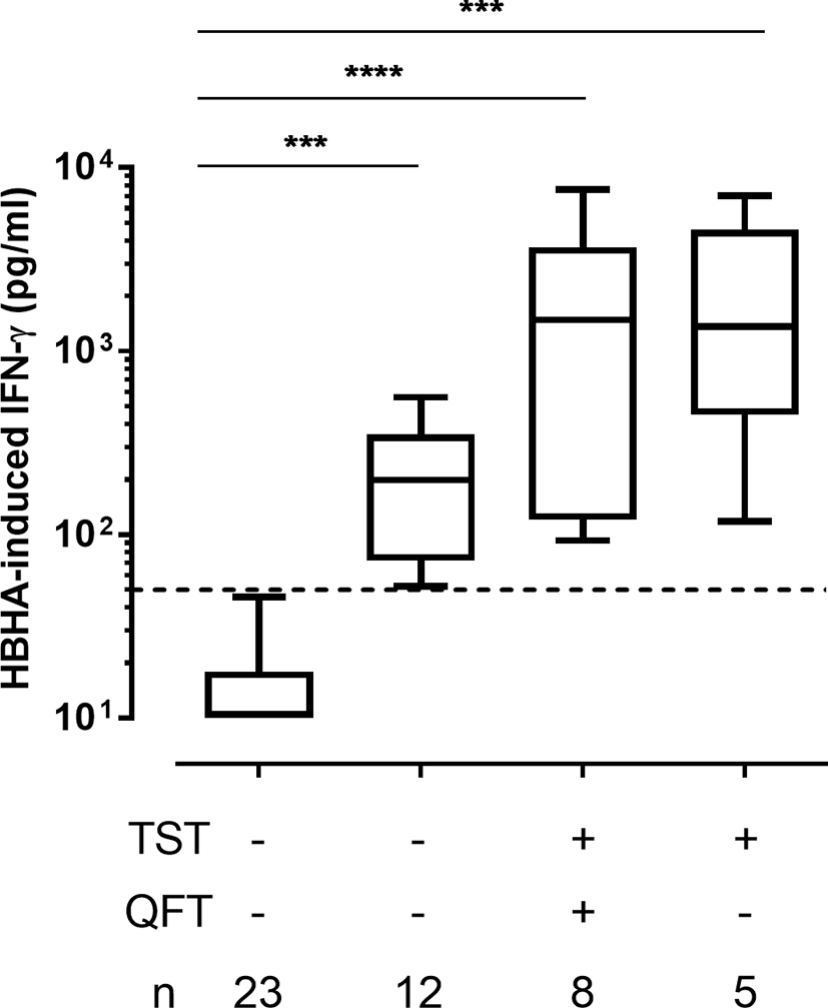
HBHA-induced- IFN-γ concentrations. IFN-γ concentrations were measured in 24 h culture supernatants of PBMC incubated with HBHA. Patients were subdivided into 4 groups according to their TST and QFT status. Boxplots represent medians and interquartile ranges (25th-75th), with whiskers (min-max). Dotted lines indicate the positivity cut-off for HBHA-IGRA. ***p ≤ 0.001; ****p ≤ 0.0001.

The HBHA-IGRA was also positive for 12/35 patients that were negative for both TST and QFT (**Figures 1** and **2B**). The demographic and clinical characteristics of these patients were not different from those of the TST^+^ patients (**Table 1**). A trend for lower HBHA-induced IFN-γ concentrations in these patients compared to the TST^+^ patients was observed, but the differences were not significant (**Figure 3**). These results indicate that within the all cohort of psoriatic patients, 51% of them have developed an IFN-γ response to the mycobacterial antigen HBHA.

### Serial HBHA-IGRA During Biotherapy

**Figure 4:**
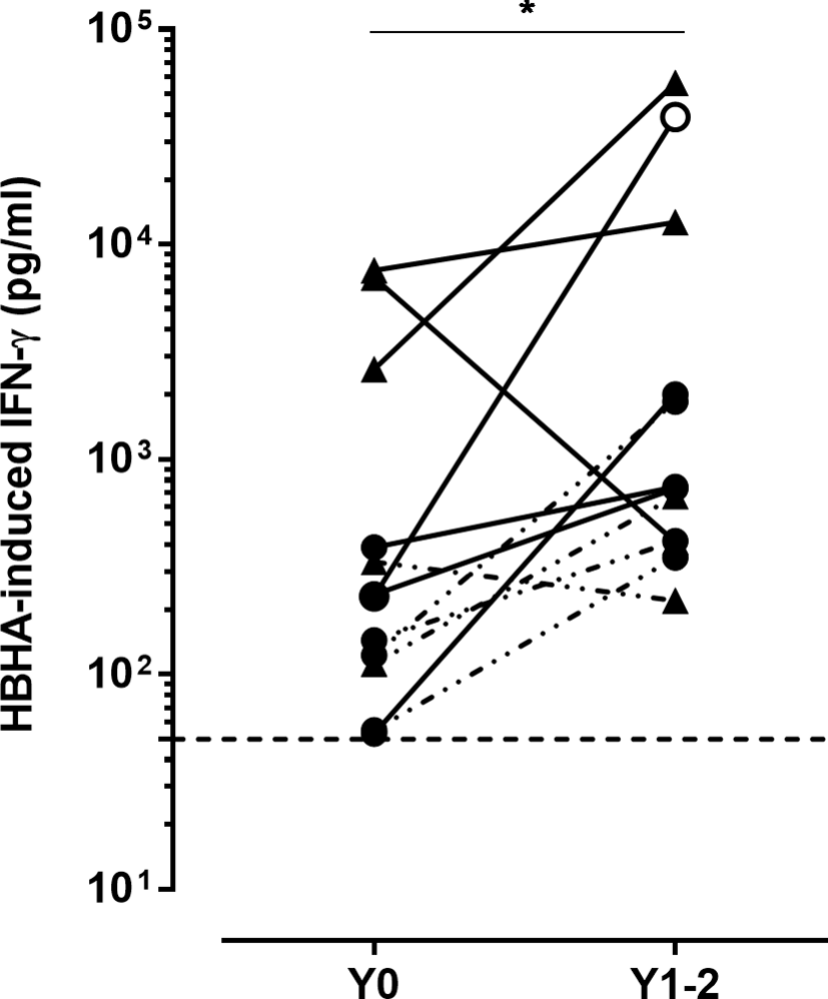
Kinetics of the HBHA-induced IFN-γ responses. HBHA-induced IFN-γ concentrations were measured in 24 h culture supernatants of HBHA-stimulated PBMC, before starting biotherapy (Y0) and 1 to 2 years later (Y1-2) in 12 patients. Triangles represent TST^+^ patients, and circles represent TST^−^ patients. Open circle represents a patient in contact with a TB index case. The lines bridge results from the same patient. Filled lines indicated patients treated with anti-TNF-α antibodies, whereas dotted lines are for patients treated with anti-IL-23 antibodies. The horizontal dotted line indicates the positivity cut-off for HBHA-IGRA. *p ≤ 0.05.

Twelve patients with a positive HBHA-IGRA were re-tested after one or two years of treatment with anti-TNF-α (n=7) or anti-IL-23 antibodies (n=5). Six of them were initially TST^+^ (with 5 QFT^+^ and 2/5 prophylactically treated for TB before starting the biotherapy), whereas the other 6 were TST^-^QFT^-^. They all were persistently positive in the HBHA-IGRA, and for 10/12 patients, the HBHA-induced IFN-γ concentrations were even higher during biotherapy than before treatment (p=0.002) (**Figure 4**). One patient, initially TST^-^QFT^-^, had a very strong increase in the HBHA-induced IFN-γ concentration between the two IGRAs (from 231 pg/ml to 39,919 pg/ml), and the QFT became positive at the second blood sampling (13.83 UI/ml) (open circle on **Figure 4**). This patient reported professional contact with a patient with aTB in the months preceding the second IGRA, so that following this contact, and after exclusion of aTB, he received prophylactic anti-TB treatment for LTBI.

### HBHA-Induced Chemokines

To further characterize the HBHA-induced immune responses in psoriatic patients, we analyzed a panel of selected cytokines/chemokines induced by HBHA in 12 HBHA-IGRA^+^ patients, and compared the results to those obtained for 3 HBHA-IGRA^-^TST^-^QFT^–^ patients included as controls. Among the HBHA-IGRA^+^ patients, 5 were TST^+^QFT^+^, 3 were TST^+^QFT^-^, and 4 were TST^-^QFT^-^ (Supplementary **Figure 1**).

**Figure 5:**
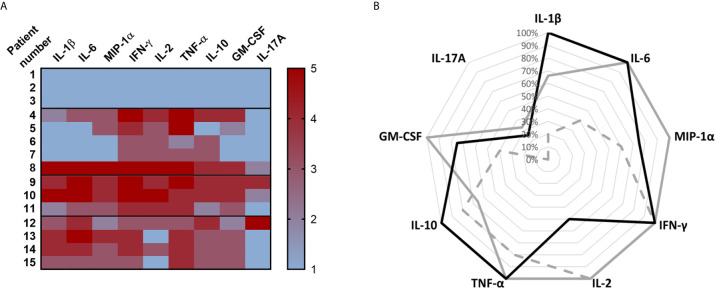
HBHA-induced cytokine/chemokine profiles. PBMC from 3 different groups of patients were *in vitro* stimulated with HBHA during 24 h and cytokines/chemokines were measured in the culture supernatants using a multiparameter based immunoassay. **(A)** Heat-map of the HBHA-induced cytokine/chemokine secretions. Three TST^−^ patients (patient n°1 to 3) were included in parallel to 5 TST^+^ QFT^+^ patients (patient n°4 to 8), 3 TST^+^ QFT^−^ patients (patient n°9 to 11) and 4 TST^−^QFT^-^but HBHA^+^ patients (patient n°12 to 15). A color scale representing the intensity of the cytokine/chemokine concentrations was established for each analyte from negative values (1, blue) to doubtful (2, pink), low and strong cytokine/chemokine concentrations (3 to 5, from light red to dark red). **(B)** Radar chart of the HBHA-induced cytokine/chemokine profiles indicating the percentages of patients within each group secreting IL-1β, IL-6, MIP-1α, IFN-γ, IL-2, TNF-α, IL-10, GM-CSF and IL-17A at concentrations higher that the upper limit of the grey zone around the defined cut-off value. TST^+^QFT^+^HBHA-IGRA^+^ patients are represented by the dotted line (n=5), TST^+^QFT^−^HBHA-IGRA^+^ patients are represented by the grey line (n=3), and TST^−^QFT^−^HBHA-IGRA^+^ patients are represented by the black line (n=4).

No HBHA-induced cytokine/chemokine was detected for the 3 psoriatic patients with an absence of identified immune response to *M. tuberculosis* (TST^-^QFT^-^HBHA-IGRA^-^) and hence considered as non-infected (**Figure 5**). In contrast, the 12 HBHA-IGRA^+^ patients were characterized by various profiles of HBHA-induced cytokines/chemokines. A restricted profile of HBHA-induced cytokines characterized TST^+^QFT^+^ psoriatic patients, compared to the TST^+^QFT^-^ patients (**Figure 5**). Most TST^+^QFT^+^HBHA-IGRA^+^ patients secreted IL-2, TNF-α and IL-10 in response to HBHA, in addition to IFN-γ, whereas the proportion of these patients secreting GM-CSF, IL-17A, IL-1β, IL-6 and MIP-1α in response to HBHA was very low (**Figure 5B**), with low concentrations of these chemokines when they were detected (**Figure 5A**). TST^+^QFT^-^HBHA-IGRA^+^ patients also secreted IFN-γ, IL-2 and TNF-α in response to HBHA, but they all additionally secreted GM-CSF, IL-6, MIP-1α, and most of them also secreted IL-10 and IL-1β, and 1/3 secreted IL-17A. All these chemokines were secreted at high concentrations (**Figure 5A**). These HBHA-induced chemokine profiles were not a consequence of psoriasis but rather reflected the LTBI status of the patients, as they share similar profiles with LTBI subjects who did not suffered from psoriasis (V.C. unpublished). Finally, all HBHA-IGRA^+^ patients in spite of negative TST and QFT secreted TNF-α, IL-10, IL-1β and IL-6 in addition to IFN-γ in response to HBHA. Most of them secreted GM-CSF and MIP-1α, and 1/4 secreted IL-17A, whereas only 50% of them secreted low concentrations of IL-2 (**Figure 5**). The profile of HBHA-induced cytokines/chemokines was thus similar in HBHA-IGRA^+^ patients who were TST^-^QFT^-^ and those who were TST^+^QFT^-^.

## Discussion

Using the recommended strategy to detect LTBI among psoriatic patients eligible to receive biological treatment, i.e. combining TST and IGRA after evaluation of the patients’ risk factors and chest X-ray results, we identified here 28.6% of LTBI psoriatic patients. This is a high proportion of LTBI patients for a low TB incidence country like Belgium where the prevalence of positive TST among healthy unexposed adolescents is 0.2% (V. Sizaire, FARES, personal communication). Among 54 adults with Crohn disease evaluated before anti-TNF-α treatment, we found only 3.7% of TST^+^QFT^+^ patients (2/54), a prevalence which remains quite low (V. Corbière, personal communication), whereas the prevalence of positive TST among TB contacts reaches 30% in Belgium (V. Sizaire, FARES – personal communication). Even if 6/50 patients mentioned a contact or a possible contact with a TB patient, the results of this study indicate that the prevalence of LTBI among psoriatic patients is elevated, in agreement with some previous reports also applying TST and/or IGRA-based guidelines for LTBI screening of psoriatic patients before anti-TNF- α treatment (15, 23). Based on TST only, 50% of patients with psoriasis who were candidates for biological therapy were treated for LTBI in Greece (23), and up to 20% in Spain (24). In these two studies, the TST cut-off level was ≥ 5 mm, which could at least partially explain the high prevalence of possible LTBI. Based on T-SPOT.TB only, 20% of psoriatic patients screened before anti-TNF-α treatment were treated for LTBI in Switzerland (25). These authors recommended to base the diagnosis of LTBI on T-SPOT.TB only rather than on TST, as most of their patients were BCG vaccinated and as they reported strong association between the T-SPOT.TB results and the presence of risk factors for LTBI (25). High prevalence of LTBI among psoriatic patients as defined by a positive TST in the Greek and Belgian studies may be attributed to possible false positive TST results due to previous BCG vaccination or immune responses to NTM. The proportion of BCG vaccinated patients in our cohort was however low (12%) as systematic BCG vaccination is not recommended in Belgium, and the proportions of TST^+^ (≥ 10 mm) attributable to BCG is very low (1%) if tested ≥ 10 years after BCG vaccination (13). Concerning a possible interference of NTM on the positivity of the TST, it remains unlikely even if it cannot formally be excluded. As nicely analyzed by Farhat et al. (13) in an extensive review of the literature and meta-analysis estimating the false positive TST results between 10 and 14 mm due to NTM, it appears that this proportion ranged from 0.1% in Montreal or France to reach a maximum of 2.3% in India (13). False-positive TST results due to immune responses to NTM in our study are unlikely as only two QFT^-^ patients had TST induration size < 15 mm (between 10 and 14 mm): one of them reported active TB history during infancy, and the other was previously treated for LTBI. All the other patients considered as LTBI had a TST induration size ≥ 15mm. Finally, we cannot formally excluded that false positive TST in psoriatic patients could occur as a result of the pro-inflammatory state of their skin (26). However, if we considered only the QFT results, the incidence of LTBI in our patients cohort reached 16% which remain higher than in the general population in Belgium. We therefore conclude that psoriatic patients evaluated for LTBI when eligible for a biotherapy are characterized by a high incidence of LTBI. As previously suggested by Ramagopolan (27), this might be due to a predisposition of patients with a past TB to develop an IMID like psoriasis as 3 patients reported a past history of TB.

As LTBI is now recognized as being an heterogeneous group of individuals with different risk of reactivation of the infection, it is widely accepted that different subgroups should be identified based on different immune responses with the aim to identify those who are most likely to reactivate the infection (10, 11). In view of the high proportion of LTBI patients detected among psoriatic patients by classical tests, this is of utmost importance within these cohorts of patients to avoid unnecessary and potentially toxic preventive anti-TB treatment. By evaluating here in addition to the QFT, the IFN-γ response to a latency-associated mycobacterial antigen, HBHA, and by analyzing also a panel of other chemokines induced by this antigen, we identified different subgroups of psoriatic patients based on their immune responses to mycobacterial antigens. The HBHA-IGRA was positive in all but one TST^+^ patients, and may therefore eventually be proposed to replace the TST, which is difficult to perform in psoriatic patients with extensive skin lesions. Among the 13 TST^+^HBHA-IGRA^+^ patients, only 8 of them had a positive QFT defining two different groups of patients with an immune response to mycobacterial antigens. The analysis of a large array of chemokines and cytokines induced by HBHA in these psoriatic patients further allowed us to substantiate the existence of two clearly distinct subgroups. Whereas TST^+^HBHA^+^QFT^-^ patients secreted several chemokines (IL-1β, IL-6, MIP-1α, GM-CSF), as well as IL-2, TNF-α, and for some of them, IL-17A, reported to play a role in protection against TB (28), TST^+^HBHA^+^QFT^+^ patients had a more restricted profile of cytokines induced by HBHA. As HBHA was reported to be a protective antigen against TB in mouse models of vaccination with HBHA followed by a challenge with *M. tuberculosis* (29, 30), and as in humans, HBHA-immune responses are more common in LTBI subjects and in treated aTB patients than in untreated patients with aTB (18, 21, 29), our results suggest that the broad array of HBHA-induced chemokines associated with a negative QFT may identify patients with a lower risk of reactivation of the *M. tuberculosis* infection. QFT^+^ patients are in contrast probably those with a higher risk of reactivation as they also have increased frequencies of *M. tuberculosis* antigens induced regulatory T cells subsets (31), known to be preferentially elevated in patients with aTB (32). Combining the results of the HBHA-induced immune responses with those of the QFT may therefore help to stratify the LTBI psoriatic patients in different subgroups and to identify patients who should be prioritized to receive prophylactic anti-TB treatment before starting biotherapy, those with a positive QFT, and not those with an isolated positive HBHA-IGRA who are better protected by their immune responses against an eventual reactivation of *M. tuberculosis* infection. This proposed attitude would have result in the prophylactic treatment of only 2/49 patients (4%) in place of 7/49 (14%) in our cohort of psoriatic patients.

We further identified a third group of psoriatic patients with positive immune responses to mycobacterial antigens. A subgroup of patients had positive HBHA-IGRA in spite of negative TST and negative QFT. Similarly to results obtained in TST^+^ patients, these HBHA-IGRA were persistently positive, often with higher responses after 1 or 2 years of biotherapy than before treatment. This suggests the existence in these patients of mycobacteria-specific memory immune responses and is consistent with a rise in intensity of IGRA responses reported previously during biotherapies (33). These HBHA-IGRA^+^TST^-^ patients had less frequent LTBI risk factors than the TST^+^QFT^+^ LTBI patients, and we cannot formally exclude a possible interference from immune responses to *M. avium* in these patients as HBHA is produced by this NTM as well (34). However HBHA proteins produced by different mycobacteria differ in their structure and activity (34), and the importance of the precise amino acid sequence and of the methylation pattern of HBHA for its recognition by T cells from LTBI subjects was demonstrated (35). Interestingly, the HBHA-induced chemokines and cytokines profiles were very similar in these HBHA-IGRA^+^TST^-^ patients to those found for TST^+^QFT^-^HBHA-IGRA^+^ patients. The induction by HBHA of IL-1β and IL-6 secretions in both TST^+^QFT^-^ and TST^-^QFT^-^ patients further suggests the possible presence in these patients of innate memory cells, as described in association with trained immunity induced by previous BCG vaccination (36, 37). These HBHA-induced immune responses do however not imply that all these psoriatic patients have an enhanced risk of TB reactivation. On the contrary, these HBHA-induced immune responses may contribute to a better protection of these patients against a reactivation or a new infection with *M. tuberculosis*. The development of LTBI (TST^+^QFT^+^) after exposure to a TB index case reported here in a psoriatic patient under anti-TNF-α treatment, having initially an immune response to HBHA with a negative TST, support this hypothesis and suggests that this patient was at least partially protected against the development of aTB disease.

We conclude that the incidence of LTBI in psoriatic patients is high, even in a low TB incidence country, and that sensitive immunological tests should be used to detect them. Combining different immunological tests may help to select patients who should be prioritized to receive prophylactic anti-TB treatment before starting biotherapies. Based on the indirect evidence of protective immune responses against aTB induced by HBHA in humans and on direct evidence in animal models, we propose that HBHA-IGRA^+^QFT^-^ patients should not be prioritized to receive anti-TB prophylaxis before anti-TNF-α treatment, but that the persistence of their protective anti-HBHA immune response during treatment should be controlled. However, more information on the predictive value of HBHA-induced immune responses for the protection against aTB development in psoriatic patients are still needed.

## Data Availability Statement

The raw data supporting the conclusions of this article will be made available by the authors, without undue reservation.

## Ethics Statement

The study involving human participants was reviewed and approved by the Comité d’éthique hospitalo-facultaire Erasme-ULB (021/406). The patients/participants provided their written informed consent to participate in this study.

## Author Contributions

FB: conceptualization, investigation, resources, writing. VD: conceptualization, data curation, methodology. FD: investigation. FW: resources. AV: data curation, investigation. CL: resources, writing. FM: conceptualization, formal analysis, funding acquisition, supervision, visualization, writing. VC: conceptualization, data curation, formal analysis, investigation, methodology, project administration, validation, writing. All authors contributed to the article and approved the submitted version.

## Conflict of Interest

The authors declare that the research was conducted in the absence of any commercial or financial relationships that could be construed as a potential conflict of interest.
