# Supplementary material for: Tuberculosis Risk Stratification of Psoriatic Patients Before Anti-TNF-α Treatment
Source: Front Immunol. 2021 Jun 3;12:672894. doi: 10.3389/fimmu.2021.672894 (PMC8209474; doi:10.3389/fimmu.2021.672894)
Supplement: Supplementary file 1 [file DataSheet_1.pdf]

Supplementary Material

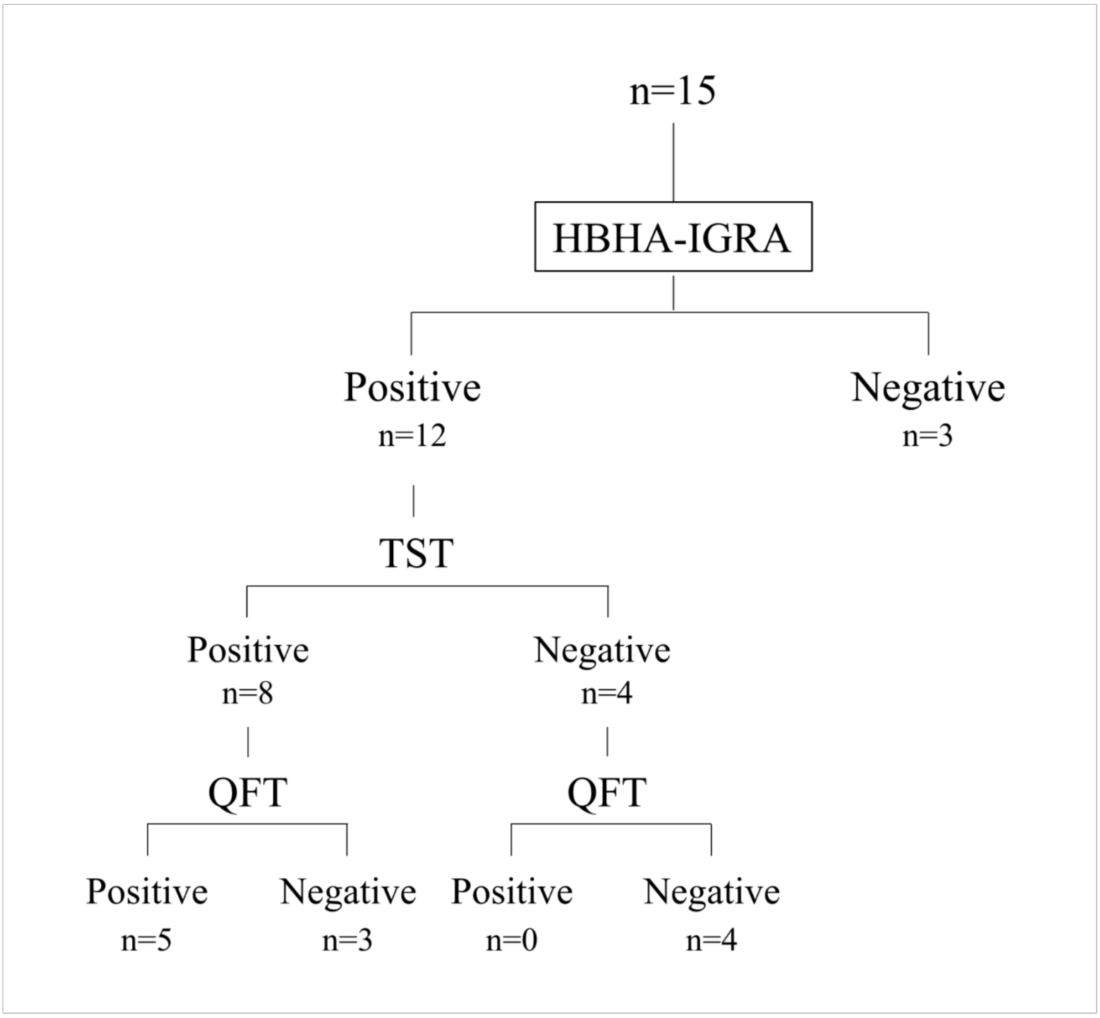

<u>Supplementary Figure 1.</u> Algorithm of the patients selected for cytokine/chemokine measurements in the HBHA-IGRA supernatants. n, number of patients; TST, tuberculin skin test; IGRA, interferon-gamma release assay; QFT, QuantiFERON-TB Gold In-tube; HBHA, Heparin-binding haemagglutinin.
